# Protective Efficacy Against Acute and Chronic *Toxoplasma gondii* Infection Induced by Immunization With the DNA Vaccine TgDOC2C

**DOI:** 10.3389/fmicb.2018.02965

**Published:** 2018-12-04

**Authors:** Nian-Zhang Zhang, Qi Gao, Meng Wang, Jun-Ling Hou, Fu-Kai Zhang, Ling-Ying Hu, Xing-Quan Zhu

**Affiliations:** ^1^State Key Laboratory of Veterinary Etiological Biology, Key Laboratory of Veterinary Parasitology of Gansu Province, Lanzhou Veterinary Research Institute, Chinese Academy of Agricultural Sciences, Lanzhou, China; ^2^Hunan Entry-Exit Inspection and Quarantine Bureau, Changsha, China; ^3^Fujian Yongcheng Agricultural and Animal Husbandry Sci-Tech Group, Fuzhou, China; ^4^Jiangsu Co-innovation Center for the Prevention and Control of Important Animal Infectious Diseases and Zoonoses, College of Veterinary Medicine, Yangzhou University, Yangzhou, China

**Keywords:** *Toxoplasma gondii*, toxoplasmosis, TgDOC2C, DNA vaccine, protective efficacy

## Abstract

*Toxoplasma gondii* is a ubiquitous intracellular apicomplexan parasite that can cause zoonotic toxoplasmosis. Effective vaccines against *T. gondii* infection are necessary to prevent and control the spread of toxoplasmosis. The present study analyzed the B-linear epitopes of *T. gondii* DOC2 (TgDOC2) protein and then cloned the C-terminus of the TgDOC2 gene (TgDOC2C) to construct the pVAX-TgDOC2C eukaryotic vector. After intramuscular injection of pVAX-TgDOC2C, immune responses were monitored. Two weeks after the last immunization, the protective effects of pVAX-TgDOC2C against acute and chronic toxoplasmosis were evaluated by challenges with *T. gondii* RH tachyzoites (genotype I) and PRU cysts (genotype II). The DNA vaccine elicited strong humoral and cellular immune responses with high levels of IgG antibody, IL-2 and IFN-γ production compared to those of the controls. The percentage of CD4+ and CD8+ T cells in mice immunized with pVAX-TgDOC2C was significantly increased compared to that of mice injected with empty pVAX I or PBS. After acute infection with 10^3^ lethal tachyzoites, mice immunized with pVAX-TgDOC2C survived longer (12.5 days) than mice treated with pVAX I (8 days) and PBS (7.5 days). Mice immunized with pVAX-TgDOC2C had significantly less brain cysts (1600.83 ± 284.61) compared to mice immunized with pVAX I (3016.67 ± 153.84) or PBS (3100 ± 246.98). Together, these results demonstrated that TgDOC2C confers protective immunity against *T. gondii* infection and may be a promising candidate antigen for further development of an effective multicomponent vaccine for veterinary use against toxoplasmosis in livestock animals.

## Introduction

Infections by the intracellular parasite *Toxoplasma gondii* are common in all warm-blooded animals, including birds and humans (Dubey, 2008; Innes, 2010; Zhou et al., 2011). If women acquire primary infection with the parasite during gestation, the fetus is at high risk of developing congenital toxoplasmosis that would manifest clinical signs of chorioretinitis, cerebral calcifications, mental retardation and hydrocephalus (Gao et al., 2012; Robert-Gangneux and Dardé, 2012; Aleixo et al., 2016). For immunocompromised individuals, reactivation of latent *Toxoplasma* infection may lead to encephalitis, ophthalmopathy and focal neurological lesions or even fatal damage (Holland, 1989; Pereira-Chioccola et al., 2009; Robert-Gangneux and Dardé, 2012; Wang et al., 2017). Toxoplasmosis in animals can cause heavy economic losses, especially in sheep and goats, arising from miscarriage, stillbirth, and neonatal death (Innes et al., 2009; Gebremedhin et al., 2014). Of more serious concern is the fact that the infected meat-producing animal is considered an important source of infection for humans (Dubey, 2009; Belluco et al., 2017; Pan et al., 2017).

Development of a safe and effective vaccine against *T. gondii* is an attractive option to prevent tissue cyst formation that can improve food safety in meat production (Zhang et al., 2013; Hiszczyńska-Sawicka et al., 2014). A DNA vaccine has elicited strong Th1-bias humoral and cellular immune responses in a mouse model to prolong the survival time after *Toxoplasma* acute infection and reduce brain cyst formation after chronic infection, which is considered a promising strategy to prevent toxoplasmosis (Gurunathan et al., 2000; Zhang et al., 2015a; Li and Zhou, 2018). Because no evaluated antigen can completely protect hosts against *T. gondii* infection, the identification of new potential vaccine candidates is a crucial step toward the development of an effective vaccine against *T. gondii* infection (Zhang et al., 2013; Li and Zhou, 2018).

Ca^2+^ is a crucial secondary messenger in the regulation of intracellular parasite attachment, invasion and egress of eukaryotic cells (Nagamune et al., 2008; Billker et al., 2009; Stewart et al., 2017). Calcium-dependent protein kinases (CDPKs) have been shown to participate in the downstream calcium-related signaling cascades as signaling mediators involved in distinct developmental processes of *T. gondii* (Billker et al., 2009; McCoy et al., 2012). TgDOC2 is another identified calcium signaling mediator that constitutes a second level of the pathway, operating downstream of CDPKs (Farrell et al., 2012; Jean et al., 2014). The DOC2 protein facilitates the membrane fusion of *T. gondii* secretory vesicles with the plasma membrane during the process of egress through regulating the Ca^2+^ signal pathway (Farrell et al., 2012). A previous study has shown that a TgDOC2-deficient strain has a disability in microneme secretion (Farrell et al., 2012). Together, these findings suggested that TgDOC2 may be a novel drug target or a vaccine candidate (Lourido and Moreno, 2015). However, there are no reports about the immunogenicity of TgDOC2 and its potential application as a vaccine candidate.

Thus, the aims of this study were to predict the potential epitopes of TgDOC2 and to evaluate its immunogenicity in the Kunming mouse model through a DNA strategy. Furthermore, the present study aimed to analyze the ability of the DNA vaccine against the infection with the highly virulent RH strain and the less virulent PRU strain.

## Materials and Methods

### Animals

Specific pathogen-free (SPF) female Kunming mice aged 6–8 weeks were obtained from the Center of Laboratory Animals, Lanzhou Institute of Biological Products (Lanzhou, China). The present study was approved by the Animal Administration and Ethics Committee of Lanzhou Veterinary Research Institute, Chinese Academy of Agricultural Sciences (Approval No. LVRIAEC2012-011). All mice used for the experiments were raised and handled in strict accordance with the Good Animal Practice requirements of the Animal Ethics Procedures and Guidelines of the People’s Republic of China.

### Parasites

Tachyzoites of the *T. gondii* RH strain (genotype I) were maintained in the State Key Laboratory of Veterinary Etiological Biology through serial passage in Kunming mice *via* intraperitoneal injection. Tachyzoites were harvested from the peritoneal exudates. The obtained tachyzoites were also used for the preparation of *Toxoplasma* lysate antigen (TLA). The PRU cysts (genotype II) were maintained monthly through oral passage in Kunming mice, and they were purified from infected brains.

### Amplification and Bioinformatics Analysis of the TgDOC2 Gene

Total RNA of *T. gondii* was extracted from the tachyzoites of the RH strain using the Trizol reagent (Invitrogen) according to the manufacturer’s protocol. The TgDOC2 gene was amplified by reverse transcription-polymerase chain reaction (RT-PCR) using the following pair of primers: C2F (5′-ATGATGAAACTAAAGGAAATGG-3′) and C2R (5′-TCAGGTGCGCCCAGCCAGATCCAGTTG-3′). The PCR product was sequenced by Sangon Biotech Co., Ltd. (China). The potential epitopes of TgDOC2 were predicted using DNAStar 8.0 (DNAStar, United States) software with the Jameson-Wolf index.

### Construction of Recombinant Plasmids

The C-terminus of the TgDOC2 gene (TgDOC2C) was amplified by PCR from the TgDOC2 gene using a pair of primers, namely, C2CF (5′-CGGGGTACCACGGAGAGAGAACGGAGAC-3′) and C2CR (5′-CCGGAATTCTCAGGTGCGCCCAGCCAGATCCAGT-3′), in which *Kpn* I and *Eco*R I restriction sites were introduced and underlined. PCR products were ligated into the pVAX I eukaryotic vector (Invitrogen, United States) through *Kpn* I/*Eco*R I sites to construct the recombinant pVAX-TgDOC2C plasmid. The concentration of extracted recombinant plasmids was determined by spectrophotometry at OD260 and OD280. The pVAX-TgDOC2C vector was diluted in sterile phosphate buffered saline (PBS) at a concentration of 1 μg/μl.

### Expression of pVAX-TgDOC2C *in vitro*

The pVAX-TgDOC2C recombinant plasmid was transfected into HEK293 cells using Lipofectamine^TM^ 2000 reagent (Invitrogen, United States) following the manufacturer’s instructions. Forty-eight hours posttransfection, the expression of pVAX-TgDOC2C *in vitro* was assayed by indirect immunofluorescence assay (IFA). In brief, pVAX-TgDOC2C-transfected cells were fixed with absolute acetone and washed with PBSTr (0.1% Triton-X-100 in PBS). Cells were then incubated with goat anti-*T. gondii* tachyzoites polyclonal antibody (in PBSTr at 1:50) for 1 h. After washing with PBSTr, cells were stained by fluorescein isothiocyanate (FITC)-labeled rabbit anti-goat IgG antibody (1:2000, Sigma, United States) for 1 h. HEK293 cells transfected with the empty pVAX I vector were treated as the negative control.

The expression of pVAX-TgDOC2C in HEK293 cells was detected by Western blot analysis. Briefly, transfected HEK293 cells (approximately 10^6^) were lysed by freezing and thawing five times, and the lysates were then subjected to SDS-PAGE. After electrotransfer, the nitrocellulose (NC) membrane (Pall, United States) was incubated with 5% bovine serum albumin (BSA) in PBST (PBS with 0.05% Tween-20) at room temperature (RT) for 1 h to block the non-specific binding sites. The membrane was washed 3 times with PBST and then was cultured with pVAX-TgDOC2C-vaccinated mouse sera (diluted in 1:500; collected at 2 weeks after the third immunization) at RT for 1 h. After washing 3 times with PBST, the membrane was incubated with goat anti-mouse IgG-HRP antibody (diluted at 1:3000, Sigma, United States) for 1 h at RT. The specific band was visualized with Clarity^TM^ Western ECL Blotting Substrates (Bio-Rad, United States) according to the manufacturer’s protocol. Mouse sera from PBS-treated mice were used as the negative control.

### Immunization and Challenge

Sixty-six female Kunming mice were randomly divided equally into three groups. Mice were intramuscularly immunized with 100 μl of pVAX-TgDOC2C and boosted with a 2-week interval. Mice treated with pVAX I vector alone or PBS served as controls. Blood samples were collected prior to each vaccination from the mouse tail vein and were centrifuged at 3,000 ×*g* for 10 min to separate blood samples. Preimmune serum samples were used as negative controls.

For evaluation of the protective effect against acute *T. gondii* infection, 10 mice from each group were intraperitoneally infected with 10^3^ tachyzoites of the virulent RH strain 2 weeks after the final immunization. The challenged mice were monitored daily until total death. In addition, another six mice from each group were orally infected with 10 brain cysts of the PRU strain as the chronic infection model. Five weeks after the challenge, the numbers of brain cysts were determined under microscopic examination.

### IgG Antibody Assay

The levels of IgG antibodies in sera from mice in each group were determined by ELISA using the SBA Clonotyping System-HRP Kit (Southern Biotech Co., Ltd., Birmingham, AL, United States) following the manufacturer’s protocol. In brief, 96-well microtiter plates were coated with 5 μg/ml TLA at 4°C overnight. After washing with PBST, plates were then treated with 1% skim milk in PBST for 1 h while shaking at RT to block non-specific binding sites. Serum (100 μl; 1:10 diluted with PBS) from each mouse was pipetted into a well and incubated for 1 h at 37°C. Each well was then incubated with 100 μl of anti-mouse IgG (HRP, 1:250 dilution, Sigma, United States) for 1 h at 37°C. Peroxidase activity was revealed by incubating with 100 μl of substrate solution (pH 4.0; 1.5% ABST, 0.03% H_2_O_2_ and 1.05% citrate substrate buffer). The reaction was stopped after the addition of 1 M H_2_SO_4_, and the absorbance was measured at 450 nm. All samples were performed in triplicate.

### Purified Splenocyte Collection

Two weeks after the last immunization, six mice per group were sacrificed to aseptically harvest the spleen. Splenocytes were obtained by filtering spleens through a nylon membrane and were purified using erythrocyte lysis buffer (Solarbio, Shanghai, China). Harvested splenocytes were resuspended in DMEM medium supplemented with penicillin/streptomycin (p/s) and 10% fetal bovine serum (FBS).

### Lymphocyte Proliferation Assay by MTS Assay

The density of purified splenocytes was adjusted into 2 × 10^5^/well in 96-well microtiter plates. Cells were cultured in DMEM medium supplemented with 10% FBS and 100 U/ml p/s. Splenocytes were then incubated with 10 μg/ml TLA or 5 μg/ml concanavalin A (ConA; Sigma, St. Louis, MO, United States) at 37°C in 5% CO_2_. Splenocytes cultured with medium alone (M) served as the blank control. The levels of proliferative responses *in vitro* were estimated using the MTS assay (Promega, Madison, WI, United States) after 92 h. The stimulation index (SI) was calculated using the following equation: OD_570TLA_/OD_570M_ or OD_570ConA_/OD_570M_.

### Flow Cytometry

CD4+ and CD8+ T lymphocytes were examined according to previous methods (Li et al., 2014). Splenocytes were first adjusted into a concentration of 10^6^ cells/ml in PBS containing 2% FBS. Staining with phycoerythrin (PE)-labeled anti-CD3 (eBioscience, San Diego, CA, United States), allophycocyanin (APC)-labeled anti-CD4 (eBioscience) and fluorescein isothiocyanate (FITC)-labeled anti-CD8 (eBioscience) was performed at 4°C for 30 min in the dark. After rinsing twice with PBS, cells were fixed in FACScan solution (1% BSA and 0.1% sodium azide diluted in PBS) and 2% paraformaldehyde. The fluorescence profiles of stained samples were analyzed using a FACScan flow cytometer (BD Biosciences) using SYSTEM II software (Coulter).

### Cytokine Assays

Purified splenocytes from mice in each group were stimulated with TLA (10 μg/ml) in 96-well flat-bottom microtiter plates. Supernatants in each well were harvested to determinate IL-4 and IL-2 levels at 24 h and IFN-γ levels at 96 h using ELISA kits according to the manufacturer’s instructions (BioLegend, United States). Briefly, the supernatants and assay buffer A were pipetted into the same well, and plates were then incubated while shaking at 200 rpm for 2 h at RT. After washing the plates four times with 1× wash buffer, 100 μl of IL-4, IL-2 or IFN-γ detection solution was added into each well and incubated at RT. After 1 h, the contents of the plates were discarded, and the avidin-HRP solution was added after washing four times and incubated while shaking for 30 min at RT. The substrate solution E was then added and incubated for 20 min in the dark. To stop the reaction, 100 μl of stop solution was added. Absorbance was read by a Model 680 microplate reader (Bio-Rad, Hercules, CA, United States) at 450 nm. Sensitivity limits for the assays were 50 pg/ml for IL-2, 20 pg/ml for IFN-γ and 10 pg/ml for IL-4. Three independent experiments were performed for analysis of the data.

### Statistical Analysis

The differences in IgG levels, CD4+ T cells and CD8+ T cells as well as the reduction in brain cysts were calculated by one-way ANOVA among at least three groups and by the *t*-test between two groups. The difference in survival time was calculated by the *Chi*-square test. All figures regarding the statistical analysis were prepared by GraphPad Prism version 5.0 (San Diego, CA, United States). A value of *P* < 0.05 was considered significant.

## Results

### Analysis of the Amplified TgDOC2 Gene

The amplified TgDOC2 gene was identified through alignment with the corresponding sequence from the http://toxodb.org/website. The cloned TgDOC2 gene showed 100% identity to the reference sequence (Gene ID: TGGT1_240910).

**FIGURE 1:**
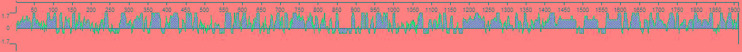
Analysis of the antigenic index of TgDOC2 protein by DNAStar 8.0 software with the Jameson-Wolf index. Linear B-cell epitopes were predicted to widely distribute in the whole TgDOC2 protein but were mainly clustered in the C-terminal.

The potential B-linear epitopes of the TgDOC2 protein was predicted using DNAStar 8.0 software. The results showed that potential B-linear epitopes of the TgDOC2 protein were mainly gathered in the C-terminal end (Figure 1). Thus, the nucleotide positions distributed between 3550 (position at 1183 aa) and the end were selected for further amplification to construct the DNA vaccine.

### Detection of pVAX-TgDOC2C Plasmid Expression in HEK293 Cells

**FIGURE 2:**
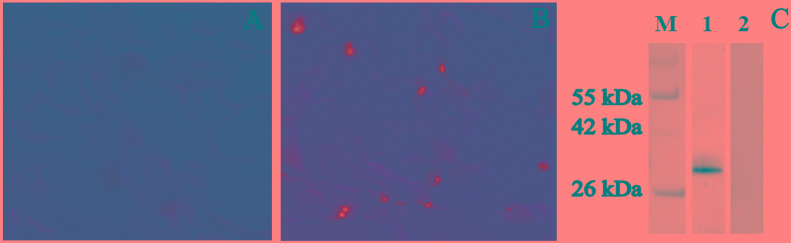
Analysis of pVAX-TgDOC2C expression *in vitro*. Expression of the pVAX I empty vector **(A)** and pVAX-TgDOC2C **(B)** in HEK293 cells was detected by indirect immunofluorescence (IFA). **(C)** The pVAX-TgDOC2C product in HEK293 cell lysate was detected by Western blot analysis. M: protein molecular weight; lane 1: membrane incubated with mouse sera from the pVAX-TgDOC2C-vaccinated group at 2 weeks after the last immunization; lane 2: membrane incubated with mouse sera from the PBS group.

The expression of pVAX-TgDOC2C *in vitro* was first examined by IFA. Green fluorescence was identified in HEK293 cells transfected with pVAX-TgDOC2C plasmids, but no fluorescence was observed in cells transfected with pVAX I alone (Figures 2A,B). These results indicated that pVAX-TgDOC2C was successfully expressed in HEK293 cells. As shown in Figure 2C, the specific band was detected in the membrane incubated with pVAX-TgDOC2C-vaccinated mouse sera but not in the membrane incubated with sera from the PBS-treated group. The size of the band was approximately 30 kDa, which was the expected size.

### Humoral Responses Induced by DNA Immunization of pVAX-TgDOC2C

**FIGURE 3:**
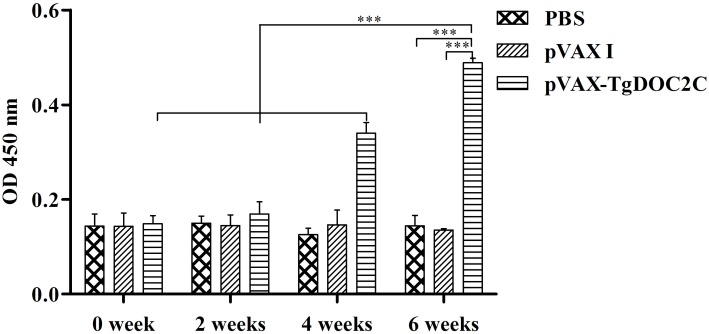
Determination of IgG antibodies in the sera of Kunming mice at 0, 2, 4, and 6 weeks. Each bar represents the mean OD (±SE, *n* = 3). ^∗∗∗^*P* < 0.0001.

The antibody levels in the sera of mice injected with pVAX-TgDOC2C increased with successive immunization and were reached the highest levels at 2 weeks after the last vaccination [*F*(3,8) = 66.21, *P* < 0.0001]. However, no differences were detected in serum samples of mice from pVAX I [*F*(3,8) = 0.04, *P* = 0.99] and PBS [*F*(3,8) = 0.27, *P* = 0.84] groups following immunization. After the third immunization, a significantly higher level of IgG antibody was examined in the sera of mice inoculated with pVAX-TgDOC2C compared to those receiving pVAX I [*t*(4) = 29.20, *P* < 0.0001] and PBS [*t*(4) = 10.78, *P* < 0.0001] (Figure 3).

### Analysis of Cellular Immune Response

As shown in Table 1, an enhanced proliferative response was detected in the splenocytes of mice immunized with pVAX-TgDOC2C after incubation with TLA [*F*(2,15) = 23.95, *P* < 0.0001] and ConA [*F*(2,15) = 4.131, *P* = 0.04] compared to those from the pVAX I and PBS groups.

**Table 1:** Splenocyte proliferative responses and the percentages of T cell subsets in immunized mice 2 weeks after the last immunization.

Groups	SI (Mean ± SD)		CD3 + CD4 + CD8 - (%)	CD3 + CD8 + CD4 - (%)
	TLA	ConA		
pVAX-TgDOC2C	1.51 ± 0.08***	1.80 ± 0.04*	13.33 ± 2.86***	8.45 ± 1.27*
pVAX I	1.32 ± 0.04	1.44 ± 0.09	3.28 ± 1.18	5.13 ± 1.84
PBS	1.30 ± 0.05	1.56 ± 0.1	3.27 ± 0.94	5.61 ± 1.11


*SI, stimulation index; TLA, *Toxoplasma* lysate antigen; ConA, concanavalin A. *n* = 6, ^∗^*P* < 0.05, ^∗∗^*P* < 0.001, ^∗∗∗^*P* < 0.0001.*

Percentages of CD4+ and CD8+ T cells were determined in spleens of mice from each group by flow cytometry analysis. The CD3+ CD4+ T lymphocytes in mice injected with pVAX-TgDOC2C were significantly higher than those in mice from the pVAX I and PBS groups [*F*(2,15) = 57.53, *P* < 0.0001] (Table 1). The ratio of CD3+ CD8+ T cells in mice of the pVAX-TgDOC2C group was higher than that in controls [*F*(2,15) = 9.3, *P* = 0.002] (Table 1).

### Cytokine Assays

Splenocytes from mice injected with pVAX-TgDOC2C had significantly higher levels of IFN-γ [*F*(2,15) = 52.66, *P* < 0.0001] and IL-2 [*F*(2,15) = 15.87, *P* = 0.0002] than those in mice in the pVAX I and PBS groups (Table 2). However, the level of IL-4 production was not significantly difference among the three groups (*P* > 0.05).

**Table 2:** Cytokine production by splenocytes of immunized Kunming mice after stimulation by toxoplasma lysate antigen^a^.

Groups	Cytokine production (pg/ml)		
	IFN-γ	IL-2	IL-4
pVAX-TgDOC2C	1584.8 474.4***	178.03 58.55**	<10
pVAX I	172.21 11.5	<50	<10
PBS	182.12 24.03	<50	<10


*^a^Splenocytes from mice were harvested 2 weeks after the last immunization. *n* = 6, ^∗∗^*P* < 0.001, ^∗∗∗^*P* < 0.0001*.

### Protective Efficacy Elicited by pVAX-TgDOC2C Vaccination

**FIGURE 4:**
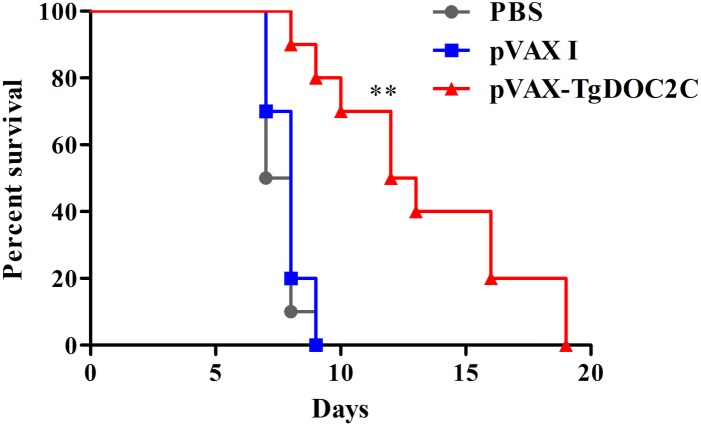
Survival rate of mice immunized with pVAX-TgDOC2C, pVAX I and PBS followed by challenge with 10^3^ tachyzoites 2 weeks after the last immunization. The mice immunized with pVAX-TgDOC2C died from day 9 to 19, which resulted in an increased survival time (12.5 days) compared to mice in the pVAX I (8 days) and PBS (7.5 days) groups (*n* = 10). ^∗∗^*P* < 0.001.

To evaluate the protective activity of the pVAX-TgDOC2C recombinant plasmid against acute *T. gondii* infection, 10 mice in each group were intraperitoneally administered with 10^3^ lethal tachyzoites 2 weeks after the last vaccination. The average survival time of mice inoculated with pVAX-TgDOC2C (12.5 days) was significantly longer than those injected with pVAX I (8 days) and PBS (7.5 days) (*P* = 0.0004) (Figure 4). Moreover, the survival times of mice in the control groups were not significantly different (*P* > 0.05).

**FIGURE 5:**
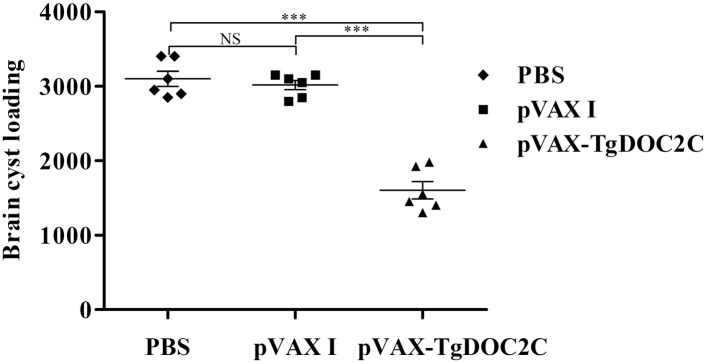
Protection against chronic toxoplasmosis in mice injected with pVAX-TgDOC2C, pVAX I and PBS 2 weeks after the last immunization. Each bar represents the mean number of brain cysts (±SE, *n* = 6). ^∗∗∗^*P* < 0.0001. NS, not significant.

The protective effect of the pVAX-TgDOC2C vaccine against *T. gondii* chronic infection was assessed through examination of brain cyst number per mouse from each group 35 days after infection of 10 cysts of the PRU strain. As depicted in Figure 5, mice immunized with pVAX-TgDOC2C (1600.83 ± 284.61) had significantly decreased brain cyst loadings compared to those injected with pVAX I (3016.67 ± 153.84) [*t*(10) = 10.72, *P* < 0.0001] and PBS (3100 ± 246.98) [*t*(10) = 9.75, *P* < 0.0001]. Immunization of mice with pVAX-TgDOC2C resulted in a cyst reduction rate of 48.36% compared to that of PBS-injected mice.

## Discussion

The present study showed that a DNA vaccination encoding the TgDOC2C gene in a mouse model promoted humoral and cellular immune responses, which contributed to a prolonged survival time (12.5 days, *P* = 0.0004) during acute infection and a decrease in brain cysts (1600.83 ± 284.61, *P* < 0.0001) during chronic infection. Significant progress has been made in recent years in the investigation of immune effects of various *T. gondii* antigens, such as ASP3 (Zhao et al., 2017), GRA2 (Ching et al., 2016), ROM5 (Zhang et al., 2015b), ROP18 (Yuan et al., 2011), ROP1 (Hiszczyńska-Sawicka et al., 2011), MIC6 (Peng et al., 2009), and SAG1 (Couper et al., 2003), and most of these antigens provide partial protection against *T. gondii* infection. The present results first demonstrated the potentiality of TgDOC2C as a vaccine candidate against acute and chronic toxoplasmosis.

DOC2, a calcium-binding protein, acts on membrane fusion and spontaneous neurotransmission in a murine model (Pinheiro et al., 2016; Ramirez et al., 2017). In intracellular parasites, the DOC2 protein impairs microneme secretion and is an essential component for exocytosis (Farrell et al., 2012; Jean et al., 2014). In the present study, the TgDOC2 protein was used to predict potential B-linear epitopes using DNASTAR software. The C-terminal end of the protein had more epitopes, suggesting that this sequence had potential to become an effective DNA vaccine against *T. gondii*. Analyses of epitopes, functions, or gene structures by emerging bioinformatics programs are widely used to design new vaccine candidates using an experimental methodology that discards non-functional sequences (Bai et al., 2012; Bastos et al., 2016; Zhou et al., 2016; Zhou and Wang, 2017). As expected, TgDOC2C is an effective vaccine candidate to improve the protective immunity against *T. gondii* challenges. Further study should design an epitope-based vaccine using calcium-dependent antigens in view of the ability of several TgCDPKs to protect mice from toxoplasmosis (Zhang et al., 2015a).

T cell-mediated immune defense is relevant to the control of toxoplasmosis (El Bissati et al., 2017), and CD8+ and CD4+ lymphocytes play a critical role during the adaptive immune response against *T. gondii* infection and reactivation (Gazzinelli et al., 1992). CD4+ T cells perform important roles in adaptive immune responses and limit intracellular pathogens in the early stage of infection (Kemball et al., 2007), while CD8+ T cells participate in the resistance in the later stage of intracellular infection through cytolytic activity (Ely et al., 1999). Enhanced CD8+ and CD4+ T cells contribute to the increasing protective immunity against *T. gondii* acute and chronic infection after DNA immunization with TgSOD (Liu et al., 2017), TgROM5 (Zhang et al., 2015b), TgeIF4A (Chen et al., 2013a) and other cocktail vaccines (Cao et al., 2015; Zhang et al., 2018). In the present study, the increased CD8+ and CD4+ T cells in mice immunized with TgDOC2C prolonged the survival time against acute infection and decreased brain cysts against chronic infection.

During the course of CD8+ and CD4+ T cell proliferation, polarization and activation, IFN-γ and IL-2 participate in certain stages and drive the immune response into Th1 type (Suzuki et al., 2011). IFN-γ is not only crucial to restrict tachyzoite growth in the early stage of infection but also important to inhibit the reactivation of dormant *T. gondii* cysts (MacMicking, 2012). Several previous studies have shown the correlation between the Th1 type immune response, characterized by the increase in IFN-γ and IL-2 production, and the enhanced resistance against toxoplasmosis in mice (Li et al., 2014; Zhao et al., 2017; Zheng et al., 2017). In the present study, significantly higher levels of IFN-γ and IL-2 production were detected in mice immunized with pVAX-TgDOC2C compared to those that received empty pVAX I and PBS, which conferred the extent of survival time after acute infection and the decrease in brain cysts after chronic infection. However, IL-4, which is associated with the Th2 immune response, was not significantly changed among the three groups. A promoted protective immunity against *T. gondii* infection along with a low secretion of IL-4 production has also been indicated in mice immunized with TgROP8 (Parthasarathy et al., 2013) and TgIF2α (Chen et al., 2013b).

The IgG antibodies against toxoplasmosis control *T. gondii* infection through inhibiting *T. gondii* duplication, facilitating the parasite attachment to immune cells and promoting macrophages to kill intracellular parasites *via* the process of antibody-dependent cell-mediated cytotoxicity (ADCC). In the present study, the increasing humoral immune response was associated with the enhanced protection against *T. gondii* infection, which was similar to the results from DNA immunization with TgROM5, TgeIF4A, TgROP18, and other antigens (Peng et al., 2009; Yuan et al., 2011; Cui et al., 2012; Chen et al., 2013a; Zhang et al., 2015b).

## Conclusion

The present study demonstrated that immunization of mice with pVAX-TgDOC2C induces a strong humoral and cellular Th1-type immune response, resulting in reduced brain cyst numbers and the extent of survival time. The vaccine potential of pVAX-TgDOC2C indicates the possibility of inclusion in further development of a multicomponent or an epitope-based vaccine against toxoplasmosis in food-producing animals.

## Availability of Data and Material

The datasets supporting the conclusions of this article are included within the article.

## Author Contributions

X-QZ and N-ZZ conceived and designed the experiments. QG, MW, F-KZ, and L-YH performed the experiments. N-ZZ and J-LH analyzed the data and wrote the paper. N-ZZ, QG, and X-QZ critically revised the manuscript. All authors read and approved the final version of the manuscript.

## Conflict of Interest Statement

The authors declare that the research was conducted in the absence of any commercial or financial relationships that could be construed as a potential conflict of interest.
